# Global comparison of phosphoproteins in human and rodent hearts: implications for translational studies of myosin light chain and troponin phosphorylations

**DOI:** 10.1186/s40064-016-2469-x

**Published:** 2016-06-21

**Authors:** K. Kotlo, A. M. Samarel, H. Y. Chen, J. Aldstadt, R. S. Danziger

**Affiliations:** Division of Cardiology, University of Illinois at Chicago, 840 S. Wood St, Chicago, IL 60612 USA; The Cardiovascular Institute and the Department of Medicine, Loyola University Chicago Stritch School of Medicine, Building 110, Rm 5222, 2160 South First Avenue, Maywood, IL 60153 USA; Division of Epidemiology and Biostatistics, University of Illinois at Chicago, Chicago, IL 60612 USA; Department of Geography, University of Buffalo, 105 Wilkeson Quad, Buffalo, NY 14261 USA; Jesse Brown Veterans Administration, 820 S. Damen, Chicago, IL 60612 USA

## Abstract

Cardiac remodeling and failure are regulated by a myriad of cardiac protein phosphorylations. In the present study, cardiac phosphoprotein patterns were examined in rodent and human hearts Left ventricular tissue samples were obtained from human systolic failing (n = 5) and control (n = 5) hearts and from two rat models of hypertensive heart failure, i.e., spontaneously hypertensive heart failure and Dahl salt-sensitive rats and corresponding controls. Phosphoproteins were separated by 2D-DIGE with Cydye staining, phosphoprotein patterns were analyzed using pixel intensity in rectified images. Specific phosphoproteins which were different in human versus rodent hearts were identified by MALDI-TOF/TOF Mass Spectrometry. Targeted pair-wise analyses showed differences (p < 0.05) in 26 % of the pixels, which included pixels containing phosphorylated troponin T, myosin light chain, peroxiredoxin, and haptoglobin. These results show differences in rodent versus human cardiac remodeling which will influence the translation rodent studies to humans in this area.

**Electronic supplementary material:**

The online version of this article (doi:10.1186/s40064-016-2469-x) contains supplementary material, which is available to authorized users.

## Background

Rodents have become a mainstay for many studies of molecular mechanisms and signaling in cardiac failure. Hypertensive rats undergo a similar morphologic progression in cardiac remodeling, including a phase of concentric hypertrophy followed by dilation and systolic failure, as observed in human hypertension.

Current evidence is overwhelming that protein phosphorylations play a key role in regulating cardiac function/remodeling and contractility in heart failure. First, a number of serine-threonine protein kinases and kinase signaling pathways have been shown to be capable of regulating features of cardiac remodeling. Among these are Phosphoinositide 3-Kinase (Cantley 2002; Shioi et al. 2000), Akt (Shiojima and Walsh 2006; Schiekofer et al. 2006; Wohlschlaeger et al. 2007). GSK-3 (Shioi et al. 2000; Matsui et al. 2002; Blankesteijn et al. 2008), transforming growth factor- (Wrana et al. 1994; Liu et al. 2006), Ca(2+)-calmodulin-dependent protein kinase (Zhu et al. 2003; Zhang et al. 2002, 2005), cAMP-dependent protein kinase (Benkusky et al. 2007; Lai et al. 2008) (Takahashi et al. 2006; Fukuda et al. 2005), Protein kinase D1 (Fielitz et al. 2005; Harrison et al. 2006), Mitogen-activated protein kinases (Frantz et al. 2007), and Protein kinase C (Agnetti et al. 2007; Sumandea et al. 2004; Sumandea et al. 2003). Second, protein phosphatases, e.g., phosphatases 1 (PP1 and PP2) (Grote-Wessels et al. 2008) (Gupta et al. 2003, 2005) (Grote-Wessels et al. 2008; Pathak et al. 2005) and Calcineurin (Dousa 1999). (Van Oort et al. 2006) (Sakata et al. 2000; Heineke et al. 2005), regulate cardiac remodeling in heart failure. Third, a number of phosphoproteins identified that may be proximal mediators of cardiac remodeling are increasing: These include Phospholamban (PLN), (Napolitano et al. 1992; Movsesian et al. 1990; Vittone et al. 2008; Altschuld et al. 1995; Schwinger et al. 1998; Desantiago et al. 2008); Connexin 43 (Akar et al. 2004; Ai and Pogwizd 2005), Endothelial nitric oxide synthase (eNOS) (Gill et al. 2005), histone deacetylase (HDAC) (Zhang et al. 2002), Protein kinase C (Vega et al. 2004), a variety of myofilament proteins (Belin et al. 2007), including troponin I (Milting et al. 2006); myosin light chain (Papp et al. 2003), and the cAMP response element binding protein (Takeishi et al. 2002; Muller et al. 1995, 1998, 2001; Matus et al. 2007); the Ryanidine Receptor (RyR) (Lehnart et al. 2005) and O transcription factor (Skurk et al. 2005; Vahtola et al. 2008). Delineation of cellular phosphoprotein signaling pathways is the result of numerous distinct studies using a wide variety experimental models, including in vitro studies with cell culture, isolated proteins, isolated cardiac myocytes, in vitro work with cardiac preparations and whole animals from numerous species and strains over the past 70 years.

In the present study, we have examined and compared phosphoprotein patterns in humans versus rodent hearts. The important question we asked in the present study is translational feasibility and efficacy of findings of rodent heart failure studies to human subjects.

The aims of the study were to compare phosphoprotein patterns (1) in human and rodent hearts and (2) between and within hearts.

## Methods

### Human hearts

*Left ventricular tissue from non*-*failing and failing human hearts.* Samples of left ventricular (LV) tissue were obtained from Loyola University Health System’s (LUHS’s) Cardiovascular Institute Tissue Repository, and from the Gift of Hope Organ and Tissue Donor Network. The investigation conformed to the principles outlined in the *Declaration of Helsinki.* A detailed protocol and informed consent document were reviewed by LUHS’s Institutional Review Board prior to tissue procurement. Following informed consent, explanted LV tissue was obtained from patients undergoing heart transplantation for nonischemic, dilated cardiomyopathy (DCM). Tissue samples were quick-frozen in liquid N_2_ in the operating room and stored at −80 °C. Following informed consent from organ donor family members, nonfailing (NF) donor hearts judged unsuitable for cardiac transplantation were stored in cardioplegic solution on ice and were delivered within 4 h of cardiac extirpation by the Gift of Hope Organ and tissue Donor Network. Tissue samples were then quickly frozen in liquid N_2_, and stored at −80 °C.

### Rodent hearts

2D-DIGE images of phosphoprotein were extracted from paired strains of failing and control rats strains, i.e., (1) Dahl salt-sensitive (S) (failing) versus resistant (R) (control) rat; and (2) Spontaneously hypertensive stroke prone (SHHF) (failing) and Wistar (control) rat strains and as previously reported and described (Kotlo et al. 2012).

### Protein preparation with phospho-enrichment

Total protein extracts were obtained by dounce homogenizing (left ventricular tissue) and resuspending pelleted regenerated cardiomyocytes in RIPA buffer containing a protease inhibitor cocktail mixture (Calbiochem) with phosphatase inhibitors (50 mM sodium fluoride, 1 mM sodium orthovanadate and 1 mM sodium pyrophosphate). The homogenate was centrifuged at 14,000 rpm for 15 min at 4 °C, and the supernatant subjected to phosphoenrichment of proteins using a Phosphoprotein purification kit supplied by Qiagen Inc according to the manufacturer’s instructions.

*2-D DIGE with Cydye staining* was performed for in-gel comparison of cardiac phosphoproteins. Phospho-enriched proteins were dissolved in 2-D lysis buffer (7 M urea, 2 M thiourea, 4 % CHAPS, 30 mM Tris-HCl, pH 8.8). Protein concentration was determined using the Bio-Rad protein assay method and samples diluted with 2-D lysis buffer to protein concentration between 5 and 8 mg/ml. 1.0 µL of diluted CyDye (1:5 diluted with DMF from 1 nmol/µL stock) is added to 30 µg of protein extract. Cy2 was used for control samples and Cy5 was used for heart failure samples. The mixture was vortexed and then incubated under dark conditions on ice for 30 min. 1.0 µL of 10 mM Lysine was added to each of the samples to quench the reaction, followed by vortexing and incubation under dark conditions on ice for an additional 15 min. Cy2 and Cy5 labeled samples were mixed together and the appropriate volume of 2X 2-D Sample Buffer (8 M urea, 4 % CHAPS, 20 mg/ml DTT, 2 % pharmalytes, and trace amounts of bromophenol blue) and 100 µL of destreak solution (GE Healthcare) was added to the samples. Rehydration buffer (7 M urea, 2 M thiourea, 4 % CHAPS, 20 mg/ml DTT, 1 % pharmalytes, and trace amounts of bromophenol blue) was added to reach a total volume of 250 µL. The samples were mixed, spun, and equal amounts of protein were loaded onto 13 cm IPG strips (pH 3–10 linear) under 1 ml mineral oil. Isoelectric focusing was performed for 12 h at 20°C with 50 µA/strip. The focused IPG strips are loaded into the 12 % SDS-gels and ran at 15 °C until the dye front ran out of the gels. Image scans are carried out immediately following the SDS-PAGE using Typhoon TRIO (Amersham BioSciences) according to the manufacturer’s instructions.

### Image registration

**Fig. 1:**
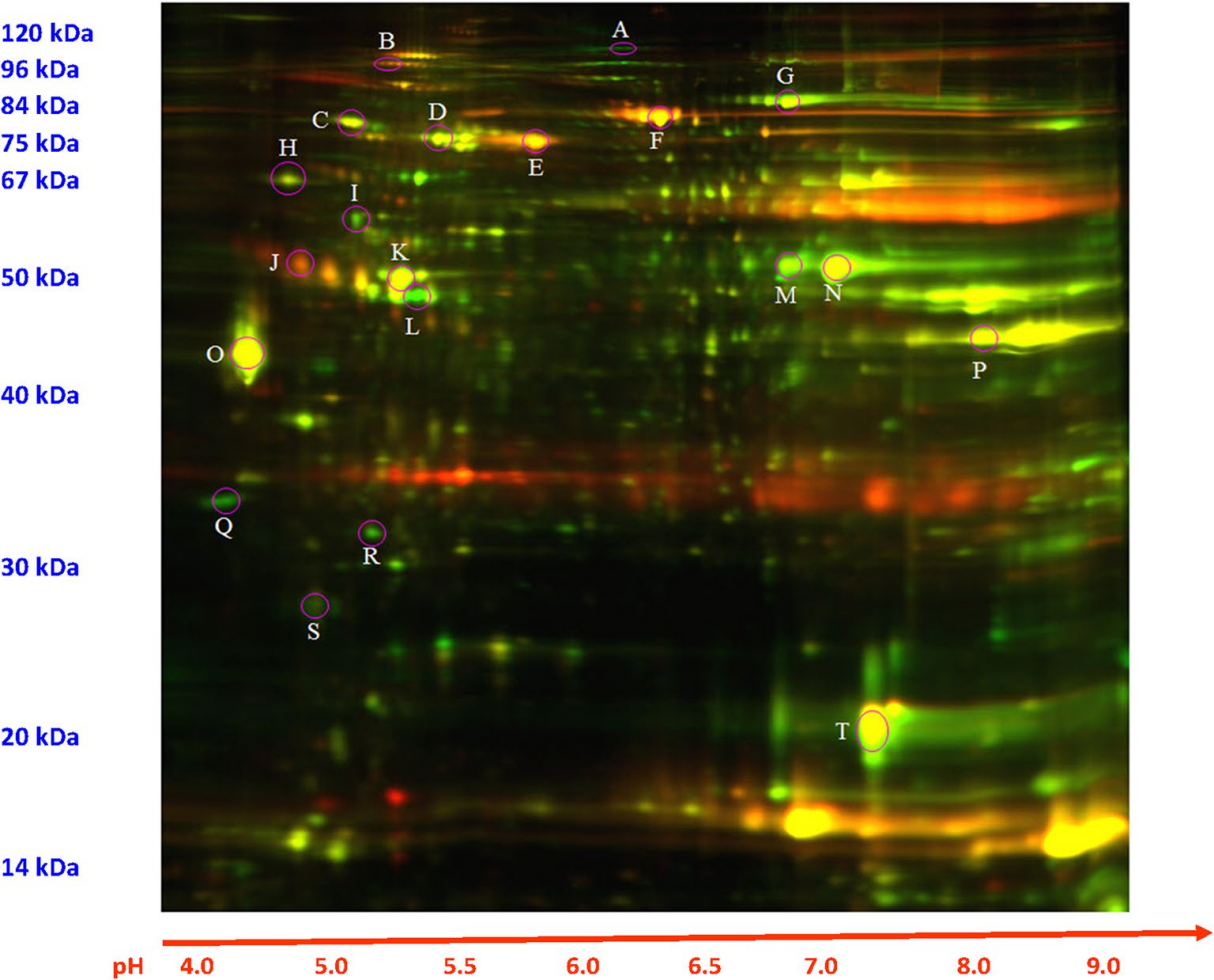
Representative 2D-DIGE image of phosphoproteins from control versus failing human heart. Rectification points are *circled* (*lettered*). Phospho-enriched protein samples from failing and control hearts were differentially labeled with Cydyes (failing = Cy5 *red*, control = Cy2 *green*) and subjected to 2D-DIGE. Yellow represents the merged spot densities. Molecular weight markers (kDa) are displayed to the *left* of each gel and pH markers are displayed underneath each gel

Scanned 2D-DIGE images were analyzed by Image QuantTL software (GE-Healthcare). The scanned gel images were saved as grayscale Tagged Image File Format (TIFF) files. Each pixel in these files had an intensity value that ranged from integers 0 to 255. Rectification points were determined by visual examination of each of the gels. Each image file was annotated with the location of 19 common points that were used for image registration (Fig. 1). All the images were transformed using spline interpolation functions available in ESRI ArcMap 10.1 software. This transformation ensures that the locations of the 19 control points are preserved exactly and the locations of pixels between the control points are transformed using a smooth function (Brown 1992). The resulting images were resampled and cropped to a box that bounds the control points. The resulting images contained 509 rows and 489 columns for a total of 248,901 pixels.

### MALDI-TOF/TOF mass spectrometry

Protein spots were excised by Ettan Spot Picker (GE Healthcare) and washed multiple times to remove staining Cy dyes and other inhibitory chemicals. Gel spots were dried and then rehydrated in digestion buffer containing sequencing grade modified trypsin. Proteins were digested in-gel at 37 °C and digested peptides are extracted from the gel with TFA extraction buffer and shaking. The digested tryptic peptides were desalted using C-18 Zip-tips (Millipore) and then mixed with CHCA matrix (alpha-cyano-4-hydroxycinnamic acid) and spotted into the wells of a MALDI plate. Mass spectra (MS) of the peptides in each sample were obtained using an ABSciex 4700 Proteomics Analyzer and ten to twenty of the most abundant peptides in each sample were further subjected to fragmentation and tandem mass spectrometry (MS/MS) analysis. The combined MS and MS/MS spectra were submitted for database search using GPS Explorer software equipped with the MASCOT search engine to identify proteins from the NCBI non-redundant protein database. Proteins identified with a confidence interval (C.I.) greater than 99 % are reported.

### Statistics

The differences between sets of images were examined on a pixel by pixel basis. Spot abundance was determined by average pixel value of “ovals” containing spots identified visually. Student’s t test was used to test for a difference in mean abundance between normal and failing human hearts and between all of the human hearts and the rodent hearts. The significance of these comparisons was assessed using the false discovery rate (FDR) (Benjamini and Hochberg 2014). R version 2.15.1 (The R Project for Statistical Computing) was employed to make the comparisons and adjust the significance values (R Foundation for Statistical Computing 2014). Since some identified phosphoproteins were of a priori high interest, these were analyzed by *t*-test (Table 2).

## Results

### Human hearts

**Fig. 2:**
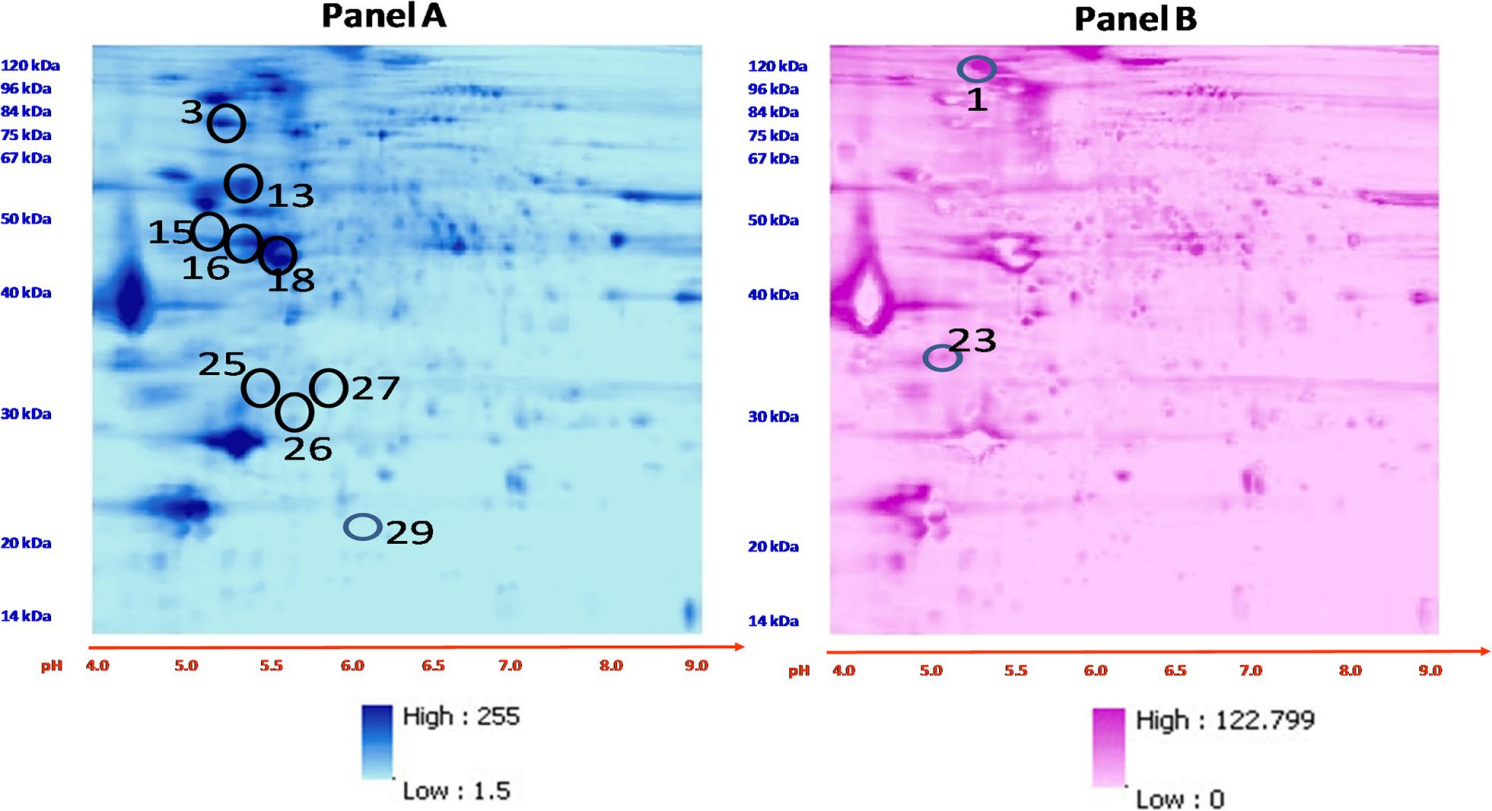
Phosphoproteins with greatest density and variability in human hearts. Analyses of composite orthorectified 2D-DIGE images of phosphoproteins extracted from human control (n = 5) and systolic failing hearts (n = 5). **a** Means of phosphoprotein spots. **b** Standard deviations of phosphoprotein spots pixels. *Color scales* to *right* of images. *Circled spots* correspond to identified phosphoproteins by MALDI-TOF (Table 2)

**Fig. 3:**
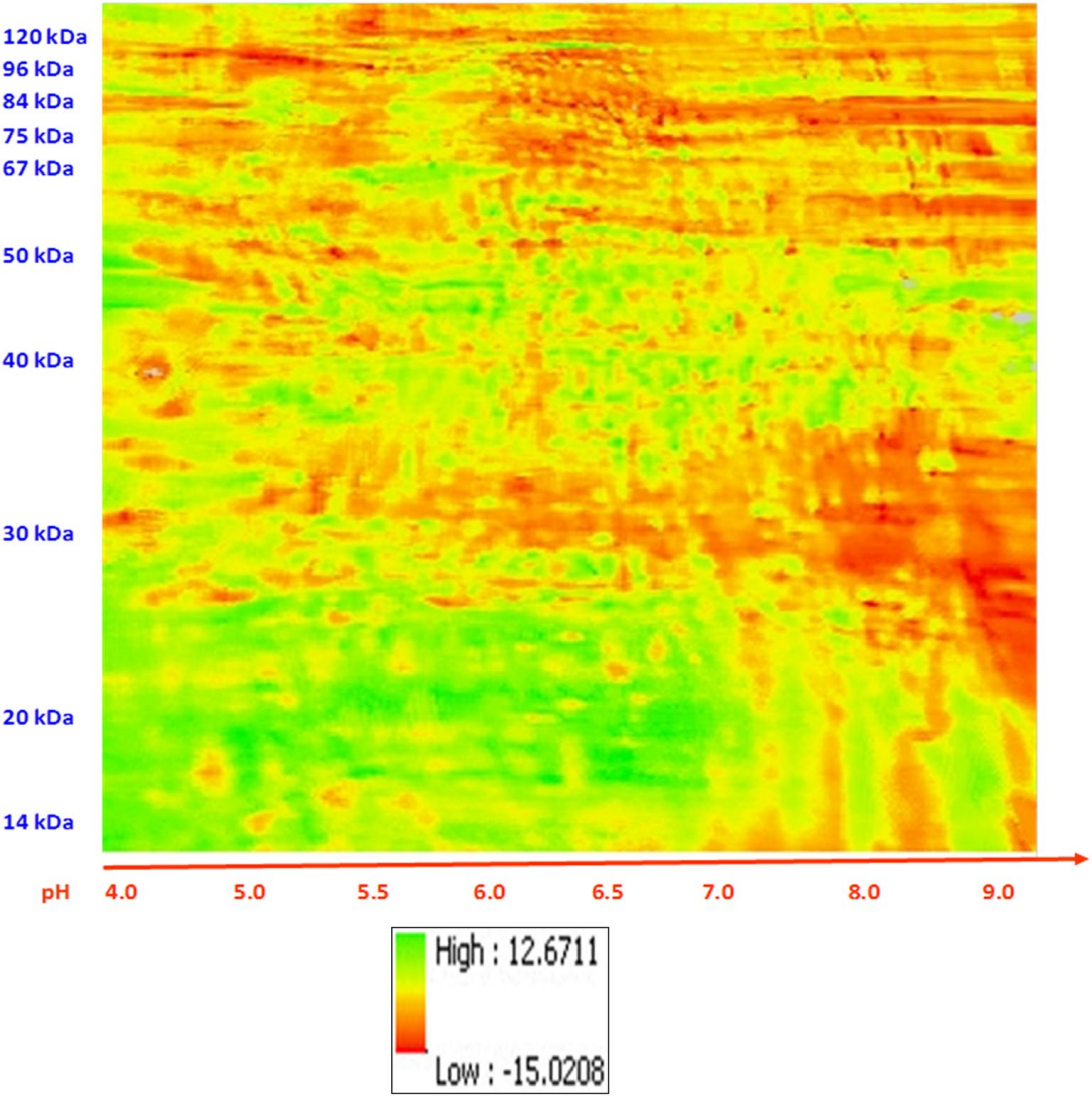
Composite orthorectified image derived from #10 2D-DIGE images of phosphoproteins extracted from human control (n = 5) and systolic failing hearts (n = 5). *Color scales* to right of images. No standard difference in means of phosphoprotein intensities of human hearts was discerned (Additional file 1)

Tissues from failing (n = 5) and control (n = 5) human hearts (Table 1) were analyzed. Phosphoproteins from each heart were separated by 2D-DIGE (Fig. 1). The distribution of these favored the right upper and lower quadrants, corresponding to proteins with MW’s 80 to 12 kDa/PI’s 6.8 to 9.0. Registered images were compiled. Mean (Fig. 2a) and standard deviation (Fig. 2b) plots showed the greatest abundance and variation in the proteins of the right upper quadrant. In identifying phosphoproteins with the greatest variability (variance in gray-unit scale) by 2D-DIGE, there is a bias toward ones with moderate abundance, in part due to censoring at extreme low or high levels. Ten phosphoproteins were chosen visually on the basis of both high abundance and standard deviation in composite images (Fig. 2a, b) for identification (Table 2) by MALDI-MS. A significant overall difference in standard differences in means of control and failing hearts (Fig. 3 and Additional file 1) could not be discerned. However, spot intensity was, in general, lowest in the lower right quadrant and greatest in lower left quadrant.

**Table 1:** Human heart samples analyzed by 2D-DIGE and MALDI-TOF

Sample ID	Age	Gender	Race	Ejection fraction	Cause of death
DCM24	55	M	Black	5 %	DCM
DCM25	45	M	White	5 %	DCM
DCM33	23	M	Black	15 %	DCM
DMC40	33	M	White	15 %	DCM
DMC41	22	F	Black	10 %	DCM
NF3	44	M	White	Echo: none done Cardiac Cath: not done	Paranoid Schizophrenia; acute subdural hematoma
NF9	50	F	Hispanic	Echo: Normal LVEF/hyperdynamic/possible mild LVH/No obvious vascular lesions, no RWMA, mild pericardial effusion Cardiac Cath: normal coronary arteries, no disease or stenosis, normal size, normal LV systolic function, EF>70 %	Left basal ganglia hemorrhagic stroke; chronic paroxysomal Afib, HTN, depression, renal insufficiency, NIDDM
NF16	40	F	Black	Echo: technically suboptimal study, RV, RA, normal dimensions; LA and LV normal dimensions, LV wall motion is normal; no gross valvular abnormality; no thrombus, vegetation or effusion; EF estimated @70 % Cardiac Cath: not done	Bipolar Disorder Suicide by methanol poisoning; seizures
NF19	61	M	White	Echo: Not done Cardiac Cath: not done	Intracranial hemorrhage
NF20	58	M	Black	Echo: Global LV systolic function normal, RV systolic functions normal, valves normal, no thrombus or effusion; EF~65 % Cariac Cath: not done	Intracranial hemorrhage, hypertension, DM

*DCM* dilated cardiomyopathy, *NF* non-failing heart (control), *EF* ejection fraction, *NIDDM* non-insulin dependent diabetes mellitus, *M* male, *F* female

**Table 2:** Phosphoprotein spots selected for greatest abundance and/or variation (SD) from 2D-DIGE composite and rectified images of human control and failing hearts (Fig. 2) and identified by MALDI-TOF/TOF mass spectrometry (CI > 99 %)

Sport number	Top ranked protein name (species)	Accession no.	Protein MW	Protein PI	Protein Count	Protein Score	Protein Score CI%	Total ion score	Total ion CI%
1	Ceruoplasmin OS = Homo sapiens GN = CP PE = 1 SV = 1	CERU-HUMAN	1,22,128	5.4	23	183	100	83	100
3	Transitional endoplascim reticulum ATP ase OS = Homo sapiens GN = VCP PE = 1 SV =	TERA_HUMAN	89,266	5.1	36	413	100	149	100
13	60 kDa heat shock rotein, mitochondrial OS = Homo sapiens GN = HSPD1 PE = 1 SV =	CH60_HUMAN	61,016	5.7	25	831	100	646	100
15	Haptoglobin OS = Homo sapiens GN = HP PE = 1 SV = 1	HPT_HUMAN	45,177	6.1	10	166	100	119	100
16	Actin, alpha cardiac muscle 1 OS = Homo sapiens GN = ACTC1 PE = 1 SV =	ACTC_HUMAN	41,992	5.2	18	701	100	562	100
18	Troponin T, cardiac muscle OS = Homo sapiens GN = TNNT2 PE = 1 SV = 3	TNNT2_HUMAN	35,902	4.9	20	755	100	574	100
23	Tropomyosin alph-1 chain OS = Homo sapiens GN = TPM 1 PE = 1 SV = 2	TPM1_HUMAN	32,689	4.7	23	521	100	339	100
25	Myosin light chain 3 OS = Homo spaiens GN = MYL# PE = 1 SV = 3	MYL3_HUMAN	21,918	5	14	316	100	214	100
27	Peroxiredoxin-2 OS = Homo sapiens GN = PRDX2 PE = 1 SV = 5	PRDX2_HUMAN	21,878	5.7	15	513	100	378	100
29	Haptoglobin OS = Homo sapiens GN = HP PE = 1 SV = 1	HPT_HUMAN	45,177	6.1	6	78	100	53	100

Spot number corresponds to Fig. 2

### Rodent hearts

**Fig. 4:**
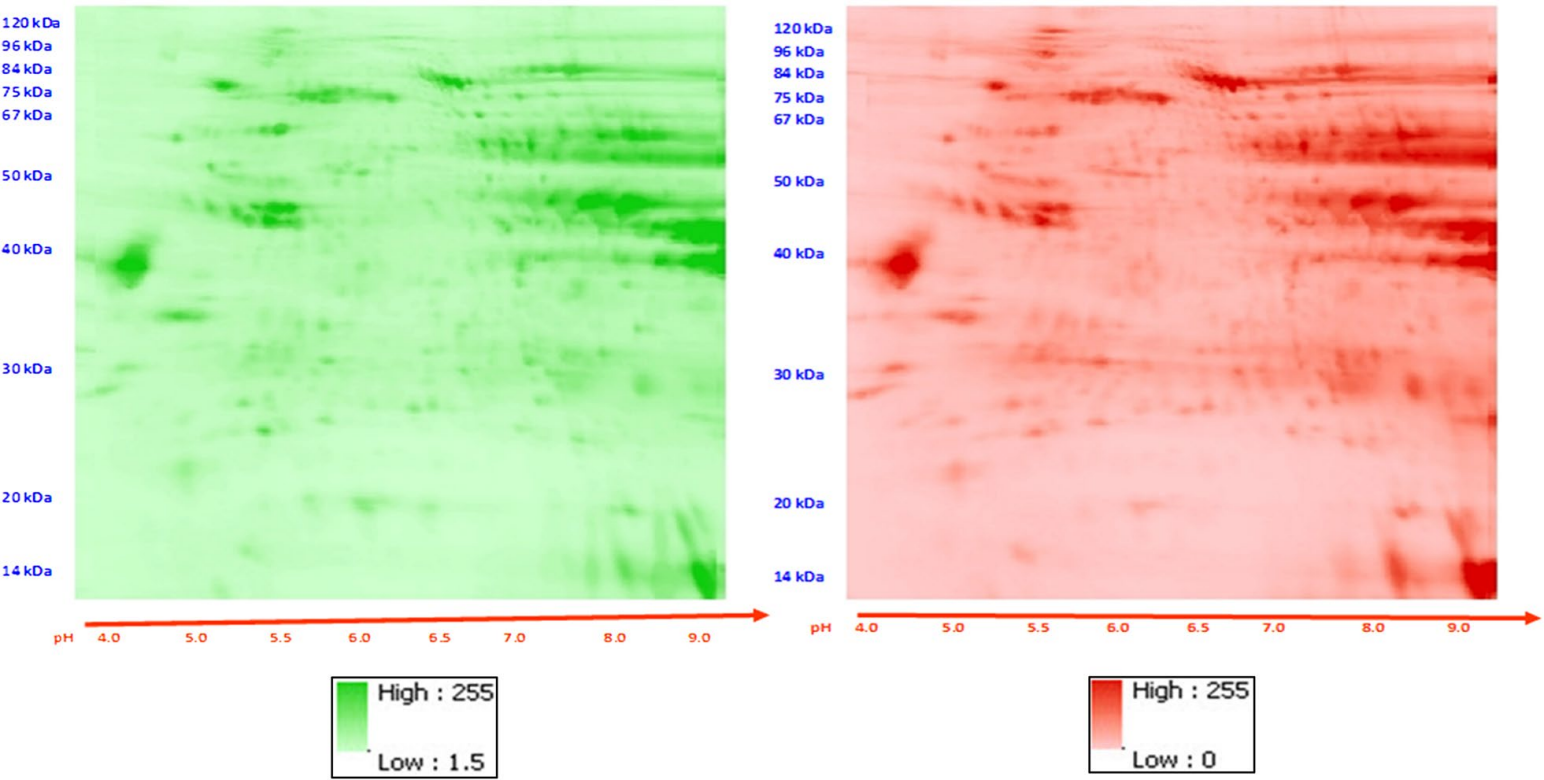
A composite orthorectified image derived from #4 2D-DIGE images of phosphoproteins extracted from control and failing rat hearts. **a** Means The *colors* represent the average difference between the controls and failing images and range from *light blue* (*turquois*) (small average differences) through *dark blue* (significant large average differences) (see *color scales*). **b** Standard deviations of means: The *colors* represent the average difference between the controls and failing images and range from *light purple* (small average differences) through *dark purple* (123) (see *color scale*) (n = 4). Greatest difference in phosphoprotein density and variability is noticed in the *upper right* quadrant

2D-DIGE images of phosphoproteins extracted from paired strains of failing and control rats strains, i.e., 1) Dahl salt-sensitive (S) and resistant (R) rat; and 2) Spontaneously hypertensive heart failure (SHHF) and Wistar rat strains (published previously (Kotlo et al. 2012)) were analyzed in composite using image registration (Fig. 4). The composite rectified images revealed a distribution of intensity of spots in all four quadrants, with the great concentration and variation in the right upper quadrant (Fig. 4).

### Comparison of cardiac phosphoproteins in humans versus rats

**Fig. 5:**
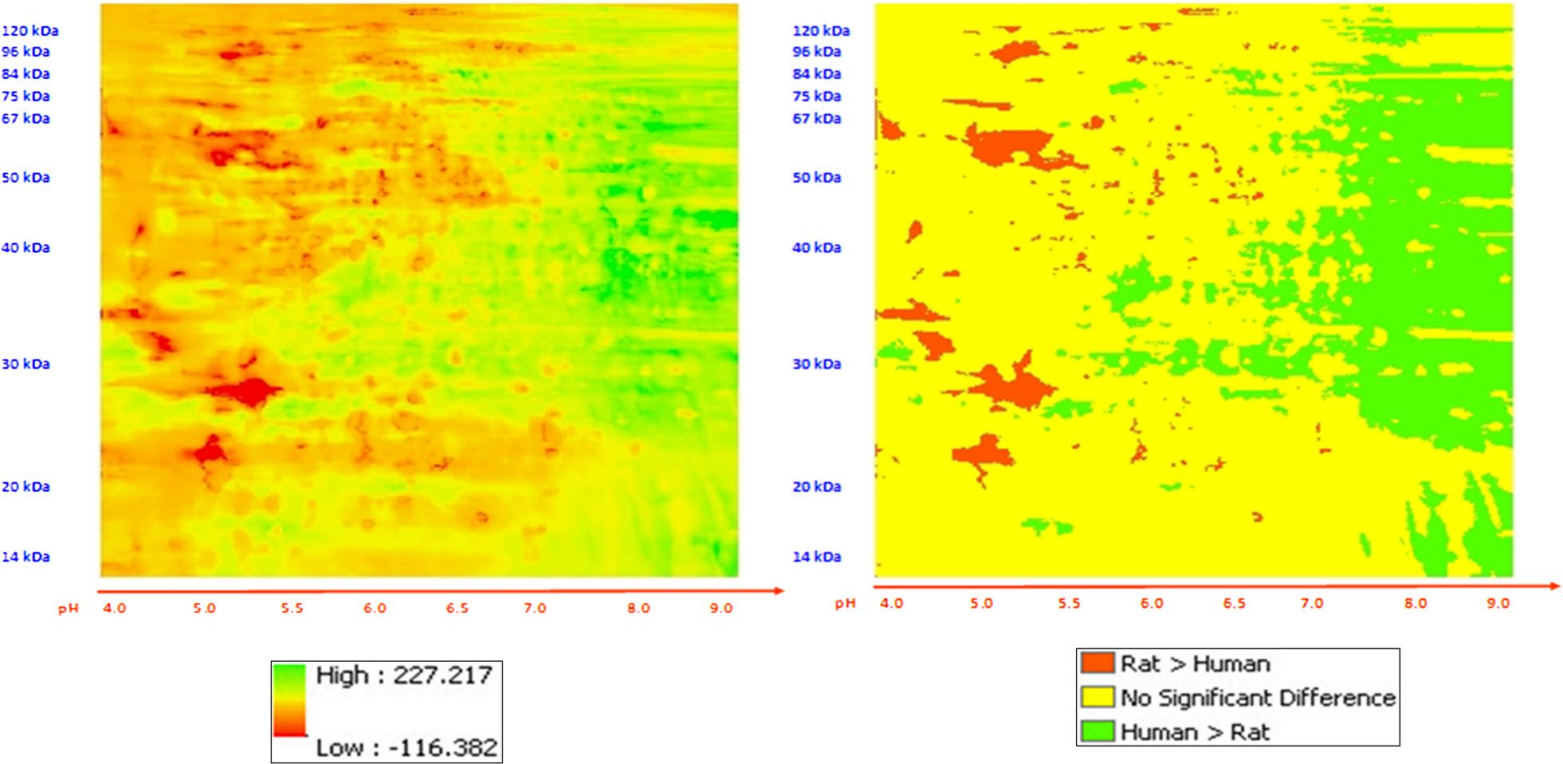
Comparison of phosphoproteins in rodent versus human hearts. **a** Standardized differences. **b** Significance map. The *bright pink areas* indicate protein phosphorylations that are different between control and failing hearts. The *light blue* (turquois) areas indicate protein phosphorylations that are most uniform across all the heart samples. rat > human—*red*; human > rat—*green*

**Fig. 6:**
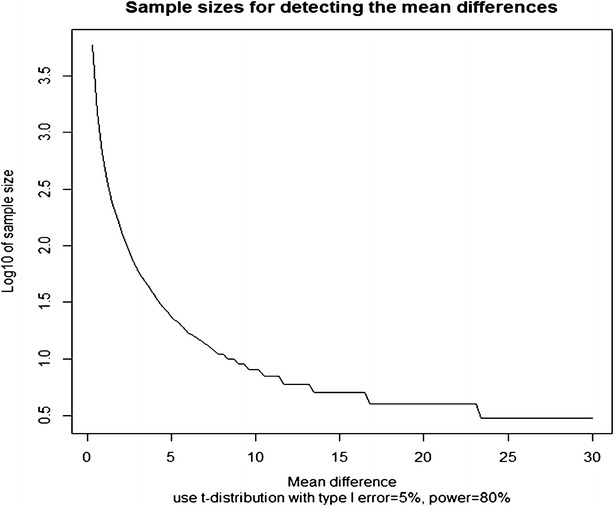
Inverse power plot demonstrating sample size required to achieve 80 % power with 5 % type 1 error for the given group mean difference based on mean and standard deviation of phosphorylated peroxiredoxin (Table 3)

The composite registered images from phosphoproteins extracted from human and rat hearts were compared (Figs. 5, 6). The range of differences in mean abundance for phosphoproteins from control versus failing human hearts was −15 to 12.67, versus a range for human vs. rat differences of −116 to 227. Mean and variance were different in human versus rodent hearts in 26 % of the image planes. Mean abundance for the humans was lower than for the rodents in focal areas in each of the four quadrants and most profound in the left two-thirds of the images. The mean pixel intensity was higher for the rat hearts in a large proportion of the right third of the images. This analysis shows statistically significant (p < 0.05) differences between cardiac phosphoproteins within combined groups of control and failing human versus rodent hearts.

The intensity spots for the identified phosphoproteins were compared between human and rodent hearts (Table 3). Specific phosphoproteins with significant differences between human versus rodent hearts were identified as 60 kDa heat shock protein (spot #13) troponin T (spot #18), Myosin Light Chain 3 (spot #25), peroxiredoxin (spot #27), and haptoglobin (spot #29).

**Table 3:** Comparison of intensity (see Methods) of phosphoprotein spots selected from Fig. 2/Table 2 for increased abundance and/or variation (SD) in human versus rodent hearts

Spot	Protein	Overall mean	Overall SD	Human mean	Human SD	Rat mean	Rat SD	t	p value
1	Cerulpolasmin	63.42	36.12	52.62	25	90.42	49.19	(−) 1.46	0.224
3	ER ATPs e	78.88	46.56	67.45	47.78	107.47	31.94	(−) 1.82	0.104
13	60kD Heat Shock Protein	84.1	32.7	93.82	32.83	59.78	17.21	2.52	0.0289
15	Haptagloblin	85.05	27.84	85.85	28.69	83.06	29.69	0.16	0.8784
16	Actin, alpha cardiac muscle 1	120.84	48.81	122.79	36.78	115.99	78.87	0.17	0.8777
18	Troponin T cardiac muscle	158.51	53.09	134.45	36.39	218.68	38.55	(−) 3.75	0.0119
23	Tropomyosin alpha-1 chain	202.96	44.58	192.67	46.69	228.7	28.9	(−) 1.74	0.1144
25	Myos in light chain 3	99.82	94.05	43.03	14.26	241.81	9.55	(−) 30.27	<0.0001
27	Peroxiredoxin-2	26.18	14.6	31.63	13.86	12.56	1.29	4.3	0.0018
29	Haptagloblin	22.23	28.72	29.04	31.74	5.21	3.36	2.34	0.0425

Spot number—Fig. 2, protein-identification—Table 2; Overall mean/SD—mean and standard deviation of intensity of spots from composite rectified 2D-DIGE images of phosphoproteins from rodent and human hearts (Fig. 3). *t*—t value for human versus rodent

## Discussion

In the present study we have applied image registration to analyze phosphoproteins separated by 2D-DIGE from multiple human and rodent control and failing hearts in composite. The central findings are that there is great biological variability in the abundance of particular phosphoproteins in human and rodent hearts.

The mean plots constructed from 2D-DIGE images show great variation in phosphoproteins in human hearts, both within and between normal and failing heart samples. There are significant differences in the standard deviations and ranges of phosphoprotein abundances, indicating significant inter-individual variation. In identifying phosphoproteins with the greatest variability (variance in gray-unit scale) by 2D-DIGE, there is a bias toward ones with moderate abundance, in part due to censoring at extreme low or high levels. Variation in the present study may not only arise from phosphorylations but also from variations in other post-translational modifications, (e.g., acetylation) which effect either MW or PI. However, the rectification and analysis process used should minimize this affecting phosphoprotein spot measurements.

We reason that the major source of variation in abundance of specific phosphoproteins observed is biological variability (versus replicative error). The overall statistical variation observed in the present study is consistent with that reported for proteomic analysis of human plasma (Corzett et al. 2010). 2D-DIGE gel to gel variability has previously been reported to be low with three or four replicates sufficient for compensation of variability, indicating that this does not introduce a significant bias with statistical confidence for individual spots (Burton and Hickey 2011). The etiology of biological variability is reasoned to arise from multiple sources, including genetic, environmental, and phenotypic cofounders. However, these may be very difficult to control for in that the variability of phosphoproteins in both rodents in the present and our previous data (Burton and Hickey 2011) is marked, suggesting that even when there is greater genetic homogeneity and control over environment, inherent variability is observed. On the other hand, since phosphoproteins have different variability in abundance, some may require smaller sample sizes to discern difference, e.g., cMyBP-C (Kooij et al. 2013).

The variability of specific phosphoproteins in human hearts may be used to infer sample sizes required for clinical trials linking a protein modification to a phenotype, e.g., heart failure. We can use the biological variability reflected in phosphoproteins in the present study to determine requisite sample sizes frequently required to link a phosphorylation to a phenotype using inverse power analyses (Fig. 6). These power analysis shows that to detect small differences, studies with 1,000 to 10,000 subjects are required, in representative phosphoproteins identified in our study. The number of phosphoproteins with high variability suggests that improved phenotyping, which is usually based on a single or few indexes, in clinical trials is unlikely to reduce the prominence of inherent biological variability reflected in cell signaling and, required sample size for statistical power.

Rodent models have become a mainstay for the study of heart disease and failure, especially with hypertension. The present results show significant differences in the phosphoprotein patterns in rat versus human hearts in approximately one quarter of the area of compared rectified 2D-DIGE composite images. Among these phosphoproteins are ones known to be important in cardiac remodeling and function as well as ones not previously linked to the heart. Among the ones linked to cardiac functions/adaptation are phosphorylated heat shock protein 60 (HSP60) and troponin T. The likelihood of significant differences in phosphorylation of Troponin T is consistent with the lack of conservation of phosphorylation motifs, e.g., troponin I Thr77 (review (Grzeskowiak et al. 2003)) in rodents versus humans. These findings may have major implications in the translation of rodent studies of the role of the phosphorylations of these proteins to humans and highlight the difficulties of such work (Hackam and Redelmeier 2006).

## Conclusions

There are significant differences in the phosphorylation of key cardiac proteins in rodents and humans, makes confirmation of cardiac signaling involving phosphorylations, e.g., kinases, phosphodiesterases, phosphatases, in rodents in humans critical for translational work. The variability of specific phosphoproteins observed indicates large sample sizes, i.e., 1000–10,000, are required to link a phenotype to a phosphoprotein.

## Additional file

10.1186/s40064-016-2469-x Cardiac phosphoproteins.

## Abbreviations

2D-DIGE: two-dimensional (2-D) difference gel electrophoresis (DIGE)
Dahl S: Dahl salt-sensitive rat strain
Dahl R: Dahl salt-resistant rat strain
MALDI-MS: matrix-assisted laser desorption/ionization mass spectrometry
SHHF: spontaneously hypertensive heart failure prone rat strain
