# Supplementary material for: Global comparison of phosphoproteins in human and rodent hearts: implications for translational studies of myosin light chain and troponin phosphorylations
Source: Springerplus. 2016 Jun 21;5(1):808. doi: 10.1186/s40064-016-2469-x (PMC4916117; doi:10.1186/s40064-016-2469-x)
Supplement: Supplementary file 1 — 10.1186/s40064-016-2469-x Cardiac phosphoproteins. [file 40064_2016_2469_MOESM1_ESM.doc]

Additional File 1 Cardiac Phosphoproteins

Table S1: Comparison of intensity of phosphoprotein spots identified and selected from Fig.2 /Table 2 for increased abundance and/or variation (SD) in failing and control human hearts. Spot number - Figure 2, protein -identification - Table 2; Overall mean/SD - mean and standard deviation of intensity of spots from composite rectified 2D-DIGE images of phosphoproteins from human failing and control hearts (Figure 3). *t* - t value for failing versus control human hearts.

| **Spot** | **Human**  **Mean** | **Human**  **S.D.** | **Control**  **Mean** | **Control**  **S.D.** | **Failing**  **Mean** | **Failing**  **S.D.** | **t** | **p-value** |
| --- | --- | --- | --- | --- | --- | --- | --- | --- |
| **1** | 52.62 | 25.00 | 42.74 | 20.36 | 62.50 | 27.33 | -1.30 | 0.2338 |
| **3** | 67.45 | 47.78 | 60.72 | 40.13 | 74.17 | 58.42 | -0.42 | 0.6840 |
| **13** | 93.82 | 32.83 | 108.21 | 40.83 | 79.44 | 15.52 | 1.47 | 0.1994 |
| **15** | 85.85 | 28.69 | 81.72 | 17.62 | 89.98 | 38.72 | -0.43 | 0.6801 |
| **16** | 122.79 | 36.78 | 138.31 | 34.76 | 107.26 | 35.12 | 1.40 | 0.1977 |
| **18** | 134.45 | 36.39 | 152.96 | 33.58 | 115.93 | 31.52 | 1.80 | 0.1100 |
| **23** | 192.67 | 46.69 | 190.66 | 58.90 | 194.67 | 37.77 | -0.13 | 0.9018 |
| **25** | 43.03 | 14.26 | 42.38 | 11.84 | 43.68 | 17.78 | -0.14 | 0.8961 |
| **27** | 31.63 | 13.86 | 31.67 | 14.15 | 31.59 | 15.23 | 0.01 | 0.9931 |
| **29** | 29.04 | 31.74 | 46.56 | 32.87 | 11.51 | 20.47 | 2.02 | 0.0845 |
